# Conservative Treatment Using Platelet-Rich Plasma for Acute Anterior Cruciate Ligament Injuries in Highly Active Patients: A Retrospective Survey

**DOI:** 10.7759/cureus.53102

**Published:** 2024-01-28

**Authors:** Shinnosuke Hada, Masao Hada, Keiichi Yoshida, Haruka Kaneko, Yoshitomo Saita, Mitsuaki Kubota, Muneaki Ishijima

**Affiliations:** 1 Department of Orthopaedics, Juntendo University, Tokyo, JPN; 2 Department of Orthopaedics, Hada Medical Clinic, Tokyo, JPN; 3 Department of Internal Medicine, Hada Medical Clinic, Tokyo, JPN

**Keywords:** accelerated return to sport, conservative treatment, anterior cruciate ligament, autologous biotherapy, platelet-rich plasma

## Abstract

Background

The recommended treatment for anterior cruciate ligament (ACL) injuries in active, sports-oriented patients is reconstructive surgery in order to regain stability and prevent secondary meniscal injuries. However, ACL reconstruction requires a long recovery period and poses issues such as decreased muscle strength due to tendon harvesting and postoperative osteoarthritis (OA) progression, thereby raising significant expectations for the advancement of conservative treatments. Recent studies have shown that platelet-rich plasma (PRP) therapy, which utilizes the tissue repair-promoting property of platelets, is effective for ACL injuries.

Methods

We administered PRP therapy within six weeks after ACL injury in patients who expressed the desire for an early return to sports through conservative care. After the treatment, patients wore a simple brace that limited deep flexion but placed no restrictions on weight bearing. Four months was the standard goal established for returning to pre-injury condition, and, depending on the target level, timing, and knee condition, we adjusted the additional PRP treatments and rehabilitation approach, gradually authorizing the patients’ return to sport. We assessed the ligament repair status by magnetic resonance imaging (MRI) just before the full return to sports. A retrospective survey was conducted to evaluate the status of ligament repair and the condition of return-to-sport in patients with ACL injuries who underwent conservative treatment using PRP.

Results

The average patient age was 32.7 years and the average treatment was 2.8 PRP sessions. MRI evaluations confirmed that ligament continuity was regained in all cases. All the patients returned to their pre-injury level (Tegner Activity Scale 7.0) in an average of 139.5 days, but there was one instance of re-rupture following the return to sports.

Conclusion

All patients with ACL injury who underwent PRP therapy regained ligament continuity and returned to sport successfully with only one case of re-rupture.

## Introduction

The treatment options for anterior cruciate ligament (ACL) injuries include surgical and conservative therapies. Conservative management of ACL injuries poses risks, such as residual instability and meniscal tears, with only about 30% of cases potentially returning to pre-injury levels of activity [1,2]. Therefore, surgical treatment is recommended for young, highly active patients, as they tend to have better outcomes in regaining activity than those undergoing conservative treatment. Conservative therapy is usually considered for patients engaged in low-impact sports and for middle-aged or older individuals [3]. However, long-term observational studies on the onset and progression of degenerative knee joint diseases (knee osteoarthritis (OA)) indicate risks associated with both conservative and surgical therapies [4].

Surgery, which involves creating bone holes in the joint, generates mechanical stress similar to intra-articular fractures that contribute to knee OA onset and progression [5]. Other issues include the lengthy rehabilitation period required for ACL reconstruction, muscle strength reduction due to tendon harvesting, and postoperative pain. In addition, considering these drawbacks, few patients may actually require reconstructive surgery, given that no complaints have been noted in 68% of ACL injury cases [6]. Currently, due to limited treatment options with established efficacy, highly active middle-aged patients, as well as young individuals and athletes desiring a quick return to sport, are left with choosing between time-consuming ACL reconstruction surgery and less successful conservative therapies. Minimally invasive surgeries enabling quicker returns and proactive conservative treatments that expedite healing are urgently needed for ACL injuries.

Traditional conservative treatments primarily involving braces and physiotherapy have not been sufficiently effective for returning to sports in pre-injury conditions. Recent reports describe the application of autologous biotherapy for ACL injuries. Specifically, platelet-rich plasma (PRP), created by centrifuging a sample of a patient’s blood to isolate the platelets, has been gaining attention [7]. The treatment depends on the ability of growth factors and anti-inflammatory cytokines contained in platelets [8] to promote the repair of damaged tissues. PRP has been increasingly administered as a conservative treatment for ACL injuries [9]. Case reports have also described effective conservative therapy using PRP in athletes playing high-impact sports at the national elite level [10].

ACL injuries are common, not just among athletes but also in recreational, middle-aged, and young individuals, including children. PRP therapy could potentially be a boon for these patients, but the applicability and limitations of this method are still unknown, requiring the continued accumulation of case studies. Furthermore, no reports have objectively evaluated ligament repair using magnetic resonance imaging (MRI).

In this study, we examined ligament continuity on MRI and return-to-sport status in 10 patients who underwent conservative treatment with PRP for ACL injury.

This article was previously posted to the Research Square preprint server on January 9, 2024.

## Materials and methods

This was a retrospective study. The study protocol complied with the principles outlined in the Declaration of Helsinki and received approval from the Research Ethics Committee, Faculty of Medicine, Juntendo University (approval number E22-0166). Due to the retrospective study design and data anonymity, the committee waived the need for informed consent.

Patients who presented with ACL injuries during the acute phase within six weeks post injury were offered several treatment options: reconstruction surgery, suture surgery, conventional conservative therapy with bracing and rehabilitation, and aggressive conservative treatment using PRP. Of these, 10 patients opted for PRP treatment (Table 1).

**Table 1:** Characteristics of the patients at time of pre-treatment F, female; M, male; BMI, body mass index; TAS, Tegnar activity score; KL, Kellgren-Lawrence grade; MMA, mixed martial arts; PRP, platelet-rich plasma Time to start PRP treatment (days): Number of days from the date of injury to the start of PRP treatment

Patient number	Age (years)	Gender	BMI (kg/m^2^)	Sports	Pre-injury TAS	Pivot shift grade	KL Grade	Time to start PRP treatment (days)
1	21	F	18.9	Cheerleading	8	3	0	15
2	18	M	21.3	Soccer	9	1	0	1
3	34	M	23.4	MMA	10	3	0	30
4	61	F	23.9	Ski	5	2	1	13
5	37	F	23.9	Ski	5	2	1	10
6	33	F	18.8	MMA	9	3	0	16
7	23	M	27.4	Rugby	9	2	0	14
8	61	M	22.1	Ski	4	1	1	39
9	17	F	20.3	Badminton	5	3	0	14
10	22	F	20.2	Volleyball	6	2	0	42

PRP was prepared using the MyCells® (Kaylight Ltd., Ramat-Hasharon, Israel) or GPS III® (Zimmer Biomet Holdings, Inc., Indiana, United States) platelet concentration systems. Peripheral blood (20~52 mL) was collected and centrifuged at room temperature to isolate approximately 3~6 mL of PRP. Based on each patient’s circumstances, PRP injections were administered into and around the joints, as necessary. Intra-articular injections were administered from the outside of the knee between the patella and femur. PRP therapy was supplemented depending on the desired outcome, timing, and knee condition. Rehabilitation involved the use of simple, commercially available braces to restrict deep bending without imposing weight-bearing limits. The rehabilitation intensity was based on the program shown in Table 2.

**Table 2:** Standard treatment and rehabilitation program

Post-treatment period	Rehabilitation and Training
0-2 Weeks	Rest; Icing; Full weight bearing; ROM:0-120°
2-4 Weeks	Upper body training; Ergometer; Walking
4-6 Weeks	Sidestep drill; Footwork
6-8 Weeks	Jogging; Skill training
8-10 Weeks	Running; Lower-body weight training
10-12 Weeks	High-intensity exercise; Full-speed running; Light-contact practice
12-16 Weeks	Return to all exercises; Full-contact practice
16- Weeks	Return to the game

Permission for a phased return to sport was adjusted according to specific goals and conditions. The efficacy of the treatment was assessed using a four-tier grading system based on MRI observations of the ligament repair status: 0 = continuous ligament with consistent low signal and thickness; 1 = continuous ligament with a high signal within; 2 = continuous ligament but thin or slack; or 3 = ligament absent or noncontinuous. Continuity was deemed achieved for grades 0-2 [11]. Rotational stability was also evaluated. Pivot-shift findings were scored as follows: 0, normal (none); 1, nearly normal (+, glide); 2, abnormal (++, clunk); or 3, severely abnormal (+++, gross). Stability was established at grade 0 (normal), or 1 (nearly normal) [12]. The final authorization for a full return to sport was granted upon the fulfillment of all the following conditions: restoration of ligament continuity on MRI, affirmation of the endpoint in the Lachman test, enhancement to grade 0 or 1 in the pivot-shift test, recuperation of muscle strength, and disappearance of patient apprehension. Of those treated, a retrospective assessment was conducted on 10 patients engaging in sports activities with a Tegnar Activity Scale (TAS) score [13] of 5 or higher. Other parameters evaluated in this study included the grade of repair on MRI, pivot-shift test results, return-to-sport status, OA assessment based on the Kellgren-Lawrence (KL) grade [14], and the emergence of complications.

## Results

**Figure 1:**
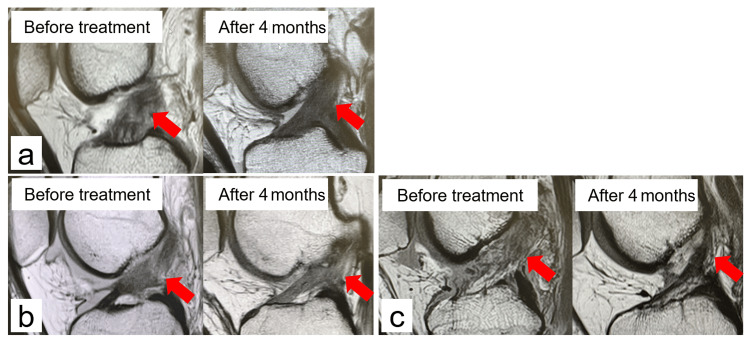
Repair condition grade assessed by MRI (a) Case 8: male 61 years old; the ACL, which was damaged and loose before treatment, regains continuity and tension four months later, and in addition, the ligament is considered to have a good grade 0 repair with low signal. (b) Case 1: female 21 years old; the ACL, which was torn before treatment, regained continuity after four months, and the repaired ACL was firm and thick but also showed high-signal areas and was considered to have a grade 1 repair. (c) Case 9: female 17 years old; the ACL, which was torn before treatment, regained continuity after 4 months, but the repaired ACL was thin and considered to have a grade 2 repair. ACL: anterior cruciate ligament

The mean (±SD) age, body mass index, and pre-injury TAS score were 32.7 (±15.6) years, 22.0 (±2.5) kg/m^2^, and 7.0 (±2.1), respectively. The mean (±SD) number of PRP treatments administered was 2.8 (±1.0). The MRI assessment of ligament repair revealed that seven cases were grade 0 (Figure 1a), two cases were grade 1 (Figure 1b) and one case was grade 2 (Figure 1c).

No cases obtained a grade 3. ACL continuity was regained in all cases (Table 3).

**Table 3:** Results after PRP treatment BMI, body mass index; TAS, Tegnar activity score; KL, Kellgren-Lawrence grade; ROM, Range of motion; PRP, platelet-rich plasma Return to sports (days): Number of days from the date of injury to return to sports.

Patient number	Number of PRP treatment	Return to sports (days)	Repair grade	Pivot shift grade	KL Grade	TAS	Follow-up (month)	Complication
1	3	118	1	1	0	8	12	
2	2	121	0	0	0	9	12	
3	5	97	0	0	0	10	25	
4	3	146	0	0	1	6	10	
5	3	154	0	0	1	9	19	
6	3	98	0	0	0	9	6	
7	3	135	0	0	0	9	62	
8	3	180	0	0	1	6	11	Limited extension ROM
9	1	179	2	1	0	5	15	Re-rupture 10 month
10	2	167	1	1	0	7	15	

The patients were divided into older and younger groups, with the older patients aged over 32.7 years. All of the patients in the older group (5/5) achieved the most favorable repair condition of grade 0. Conversely, only two of the five cases in the younger group achieved grade 0. Performance in the post-treatment pivot-shift test revealed a grade 0 in seven cases and grade 1 in three cases. No cases received grade 2 or 3. All patients returned to the pre-injury performance level within a mean (±SD) of 139.5 (±29.3) days. Re-rupture occurred after returning to sport in a young woman who had only undergone one round of PRP treatment, obtained the worst repair condition on MRI among all patients of grade 2, and a pivot-shift test of grade 1. Knee OA changes at the time of injury were Kellgren-Lawrence (KL) grade 0 in seven cases and grade 1 in three cases. No cases progressed in KL grade after treatment. Regarding complications, one patient presented with a residual range of motion restriction, which subsequently improved with local anesthetic injection treatment.

Representative case

**Figure 2:**
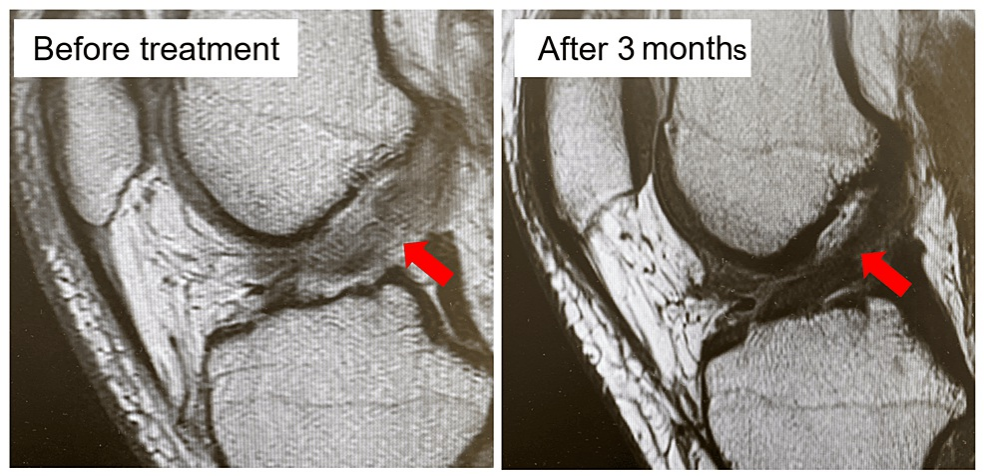
Magnetic resonance imaging of the representative case Case 6: female 33 years old The anterior cruciate ligament, which was torn and disconnected before treatment, has regained continuity three months later, and furthermore, the intra-ligament is considered to have good grade 0 repair with low signal.

A 33-year-old female mixed martial arts (MMA) fighter had a pre-injury TAS score of 9. During training, she twisted her right knee severely while receiving an attack from an opponent and consulted the clinic 16 days later. The Lachman test showed a disappearance of the endpoint, and the pivot-shift test was grade 3. Joint aspiration obtained 30 cc of bloody effusion. MRI showed a rupture in the central part of the ACL (Figure 2).

Surgical reconstruction was offered by the previous physician and was deemed the inevitable option due to the demands and level of the patient’s chosen sport. Having had a history of ACL reconstruction surgery for a left ACL injury and having consequently undergone a long recovery period, the patient wanted an early return through conservative therapy using PRP. PRP treatment was administered thrice at two, three, and five weeks after the injury. In the sixth week, an endpoint appeared on the Lachman test, and jogging was advised. At 10 weeks, the patient’s fear of movement disappeared, and her pivot-shift test was grade 1. Thus, she was permitted to return to training. The patient returned to competition at 12 weeks, and her TAS score recovered to the pre-injury level of 9. An MRI 12 weeks after the injury clearly visualized an intact ACL (Figure 2). At this point, the pivot-shift test was grade 0. The patient continued to compete without any re-injury or the knee giving way until the final follow-up observation.

## Discussion

All 10 patients with ACL injuries in the current study reacquired ligament continuity on MRI and returned to their pre-injury sport level within an average of 139.5 days. Seven patients achieved a grade 0 ligament repair score. Previous data have shown that conservative treatment restores ligament continuity in 58% of patients, with 33% of patients achieving a grade 0 ligament repair score. Thus, the repair rate in the present study was relatively good.

Both fundamental and animal studies have demonstrated that platelets promote healing in ACL injury through anti-inflammatory cytokines that suppress initial inflammation and various growth factors, such as platelet-derived growth factor (PDGF), transforming growth factor-β (TGF-β), vascular endothelial growth factor (VEGF), and insulin-like growth factor 1 (IGF-1). These components stimulate mesenchymal cells and fibroblasts and promote extracellular matrix synthesis [7,8,15,16]. Furthermore, the addition of PRP to grafts after ACL reconstruction surgery can shorten graft maturity by 48% [17] and potentially lower the re-rupture rate by one-third [18]. The growth factors in PRP may facilitate graft maturation, accelerate the natural remodeling process, and promote a shorter and stronger maturation process, thereby improving both the duration and quality of the healing process [19,20]. While the ends of a torn ACL may remain somewhat in contact during the acute phase, they are absorbed over time, gradually losing contact and eventually adhering to the posterior cruciate ligament or intercondylar area or otherwise completely vanishing. However, the introduction of PRP during the acute phase when the ends are still in contact may promote ligament healing and reacquisition of continuity [21,22].

One significant issue with ACL surgical reconstruction is the long rehabilitation period required before returning to sport, usually around six to nine months [3]. However, in this case series, the time to return to sport was an average of 139.5 days, with the shortest case being 97 days and the longest 180 days, thereby demonstrating the possibility of an early return. While conventional conservative therapy presents approximately a 30% chance [2] of returning to the pre-injury level, all 10 cases in this series achieved this benchmark. Similar clinical reports have shown that an average of 81% of high-level professional soccer players with ACL injuries who underwent PRP therapy were able to return to their pre-injury performance levels within 16 weeks [22]. Furthermore, reports have even described successful treatment among athletes engaged in high-impact sports, such as rugby and MMA [10]. In the present study, one patient experienced re-rupture after returning to sport.

The re-rupture rate for ACL surgical reconstruction is approximately 1.8-10.4% [1], similar to the rate obtained in the present case series. The one re-rupture occurred in a young female patient who received PRP only once and obtained a ligament repair score of grade 2. Cases with ligament repair grade 0 or 1 on MRI tend to have better outcomes and functionality than those with grade 2 or 3 [13], which suggests the possibility of insufficient graft repair in the present re-rupture case. ACL surgical reconstruction has a poorer outcome in younger patients [1]. However, a similar trend was seen in the present case series, with fewer cases repaired to grade 0 in the younger group, indicating the need to improve outcomes in younger patients. Younger patients may need to receive PRP treatment earlier and more frequently and to proceed cautiously with rehabilitation. To increase the success rate of treatment in the future, more cases need to be evaluated to conclusively identify the factors affecting treatment results and clarify indicators and timing for possible return to sport.

While PRP was administered through intra-articular injection in this study, some reports describe other methods, such as directly injecting into the ACL under ultrasound guidance [9]. Nevertheless, discussions on the best method of administration are needed moving forward. Long-term issues following ACL injury include the onset and progression of knee OA, with 90% of cases progressing to OA within 20 years [23]. Notably, the incidence of OA at 12 years post treatment is similar, whether after ACL reconstruction or conservative treatment [24]. Thus, preventing OA progression after ACL injury is a crucial long-term therapeutic interest. Reasons for OA progression after ACL injury or reconstruction include the activation of intra-articular protease mechanisms, such as a disintegrin and metalloproteinase with thrombospondin motifs due to inflammatory changes during injury [25], secondary damage to cartilage and meniscus due to residual joint instability [3], chondrocyte apoptosis caused by trauma similar to intra-articular fractures from creating bone holes during ACL reconstruction [26], and promotion of osteophyte formation due to excessive TGF-β production [27,28]. Although OA changes appear relatively early within a year after ACL reconstruction [6], no progression in KL grade was observed in the present case series, and no onset of radiographic OA was noted in the short term. Successful conservative treatment with PRP therapy could prevent joint degeneration due to its anti-inflammatory effects at the time of ACL injury, secondary cartilage and meniscus damage through stability reacquisition by ACL repair, and intra-articular damage by averting surgery and thus potentially suppressing OA progression through the associated complex factors.

In the present study, all 10 patients were able to return to their pre-injury levels quickly and in good condition, with good ligament repair, as confirmed by MRI. Our findings indicate PRP therapy as a promising option in the active conservative treatment of ACL injuries. The limitations of the present study are that the results are based on only 10 cases and that it is a retrospective study. Thus, the evaluation of more case is required, and large-scale comparative studies on surgical treatment and conventional conservative treatment are essential.

## Conclusions

Ten highly active patients with acute ACL injuries were treated conservatively with PRP therapy. All patients had restored ACL continuity and were able to return to their pre-injury activity. In seven patients, the ACL was excellently repaired on MRI, and re-tears occurred in one patient with inadequate repair. None of the patients had advanced knee OA grade.
